# “You need a team”: perspectives on interdisciplinary symptom management using patient-reported outcome measures in hemodialysis care—a qualitative study

**DOI:** 10.1186/s41687-022-00538-8

**Published:** 2023-01-20

**Authors:** Brigitte Baragar, Kara Schick-Makaroff, Braden Manns, Shannan Love, Maoliosa Donald, Maria Santana, Bonnie Corradetti, Juli Finlay, Jeffrey A. Johnson, Michael Walsh, Meghan J. Elliott

**Affiliations:** 1 Department of Medicine, Cumming School of Medicine, University of Calgary, TRW Building, 3280 Hospital Drive NW, Calgary, AB T2N 4Z6 Canada; 2 Faculty of Nursing, University of Alberta, Edmonton, AB Canada; 3 Department of Community Health Sciences, University of Calgary, Calgary, AB Canada; 4 O’Brien Institute of Public Health, University of Calgary, Calgary, AB Canada; 5 Department of Pediatrics, University of Calgary, Calgary, AB Canada; 6 Medicine Strategic Clinical Network, Kidney Health Section, Alberta Health Services, Edmonton, AB Canada; 7 School of Public Health, University of Alberta, Edmonton, AB Canada; 8 Department of Medicine, McMaster University, Hamilton, Canada; 9 Department of Health Research Methods, Evidence and Impact, McMaster University, Hamilton, Canada; 10 Population Health Research Institute, Hamilton Health Sciences / McMaster University, Hamilton, Canada

**Keywords:** Qualitative research, Kidney failure, Hemodialysis, Patient-oriented research, Patient-centered care, Patient-reported outcome measures

## Abstract

**Background:**

Patient-reported outcome measures (PROMs) are standardized instruments used for assessing patients’ perspectives on their health status at a point in time, including their health-related quality of life, symptoms, functionality, and physical, mental, and social wellbeing. For people with kidney failure receiving hemodialysis, addressing high symptom burden and complexity relies on care team members integrating their expertise to achieve common management goals. In the context of a program-wide initiative integrating PROMs into routine hemodialysis care, we aimed to explore patients’ and clinicians’ perspectives on the role of PROMs in supporting interdisciplinary symptom management.

**Methods:**

We employed a qualitative descriptive approach using semi-structured interviews and observations. Eligible participants included adult patients receiving intermittent, outpatient hemodialysis for > 3 months, their informal caregivers, and hemodialysis clinicians (i.e., nurses, nephrologists, and allied health professionals) in Southern Alberta, Canada. Guided by thematic analysis, team members coded transcripts in duplicate and developed themes iteratively through review, refinement, and discussion.

**Results:**

Thirty-three clinicians (22 nurses, 6 nephrologists, 5 allied health professionals), 20 patients, and one caregiver participated in this study. Clinicians described using PROMs to coordinate care across provider types using the resources available in their units, whereas patients tended to focus on the perceived impact of this concerted care on symptom trajectory and care experience. We identified 3 overarching themes with subthemes related to the role of PROMs in interdisciplinary symptom management in this setting: (1) Integrating care for interrelated symptoms (*“You need a team”*, *conducive setting, role clarity and collaboration*); (2) Streamlining information sharing and access (*symptom data repository*, *common language for coordinated care*); (3) Reshaping expectations (*expectations for follow-up*, *managing symptom persistence*).

**Conclusions:**

We found that use of PROMs in routine hemodialysis care highlighted symptom interrelatedness and complexity and helped to streamline involvement of the interdisciplinary care team. Issues such as role flexibility and resource constraints may influence sustainability of routine PROM use in the outpatient hemodialysis setting.

**Supplementary Information:**

The online version contains supplementary material available at 10.1186/s41687-022-00538-8.

## Plain English summary

People with kidney failure receiving hemodialysis are faced with complex symptoms that impact their day-to-day functioning and quality of life. Patient-reported outcome measures (PROMs) are tools used by patients to directly communicate symptoms to their care team and guide symptom-focused care. Little is known about how PROMs could be integrated into the team-based care models of outpatient hemodialysis centres. In this study, we conducted interviews with people receiving hemodialysis and their clinicians about their perspectives on how PROMs could support interdisciplinary symptom management (i.e., integration of expertise to achieve common management goals). Participants described how the interrelatedness of symptoms was well suited to an integrated care approach and how PROMs enhanced communication and access to information across team members. In cases where symptoms persisted despite appropriate treatment, patients and clinicians explained how PROMs served as a tool to set realistic goals and reshape illness perception. Findings from this study suggest that access to resources, role flexibility, and established relationships within hemodialysis centres are important for sustaining PROM use in this setting.

**Supplementary Information:**

The online version contains supplementary material available at 10.1186/s41687-022-00538-8.

## Introduction

Patients with kidney failure receiving hemodialysis face high symptom burden and associated challenges in managing symptoms alongside their dialysis, comorbidities, and other day-to-day demands [1]. Symptom burden can contribute to morbidity, high healthcare use, and low health-related quality of life [1–3], the latter of which is often underappreciated by the dialysis healthcare team [4, 5]. In a national priority-setting exercise, patients with kidney failure and their healthcare providers identified maintaining symptom control, level of functioning, and wellbeing as a top priority [6]. The increasing global prevalence of kidney failure further underscores a need for systematic and patient-centered approaches to enhance symptom detection and care delivery in this population [7].

Patient-reported outcome measures (PROMs) are standardized instruments for assessing patients’ perspectives on their health status at a point in time, which encompasses their health-related quality of life, symptoms, functional status, and wellbeing in physical, mental, and social aspects of health [8–10]. As reports coming directly from patients about how they function and feel, PROMs can bridge discordances between providers’ beliefs about patients’ health and their lived experience [5, 11]. PROMs have been used widely in clinical effectiveness research to track symptoms and health-related quality of life and as benchmarking tools to improve quality of care, but until recently they have been underutilized in the clinical hemodialysis context [12]. Ideally, PROMs would be completed by patients and their results fed back to clinicians, who would use that information to guide patient care [13, 14]. Studies in other clinical areas using PROM reports to direct care suggest improved patient-provider communication, better health-related quality of life, and, in some cases, lower mortality [15–19]. Although some kidney care jurisdictions have mandated routine collection of PROMs, optimal approaches for integrating them into hemodialysis care and their impact on health outcomes remain unclear [12].

Interdisciplinary care models provide coordinated, integrated, and patient-centered care across separate disciplines to achieve common management goals [20]. Whereas comprehensive clinics that engage nursing, medical, and allied health professionals have become commonplace for individuals with advanced, non-dialysis-dependent chronic kidney disease [21–23], how care is integrated across disciplines and how tools such as PROMs might enable concerted care in the hemodialysis setting are not well understood. Alongside a program-wide initiative integrating PROMs into routine hemodialysis care across Southern Alberta, Canada [24], this study aimed to explore patients’ and clinicians’ perspectives on the role of PROMs in supporting interdisciplinary symptom management.

## Methods

### Study design and setting

This qualitative study was embedded within a pragmatic, cluster randomized controlled trial, Evaluation of routinely Measured PATient reported outcomes in HemodialYsis Care (*EMPATHY*) described elsewhere [25]. The initiative rolled out across 3 geographic areas in Canada and assessed the impact of bi-monthly screening of patients using PROMs paired with treatment guides (i.e., clinician- and patient-specific resources and handouts with suggested management approaches) on patient-clinician communication, clinical outcomes, and healthcare utilization compared with usual care [24]. This qualitative study took place across the 7 participating hemodialysis units assigned to an intervention group, where patients completed the Integrated Palliative Outcome Score [IPOS]-Renal, EQ-5D-5L, or both depending on unit allocation [26, 27]. Upon PROM completion, a personalized symptom report was generated that displayed responses using a visual ‘stoplight’ system. Bedside nurses were responsible for reviewing this report with patients, determining which concerns patients wanted to address, and referring to the patient- and clinician-directed treatment guides to initiate and escalate management.

We used a qualitative descriptive methodology to provide insight into how patients and healthcare providers perceive the role of PROMs in interdisciplinary team-based hemodialysis care in the context of this initiative [28, 29]. Qualitative description enables rich, descriptive accounts of individuals’ experiences, perspectives, and insights while remaining close to the data [30]. We selected this methodology as it offers a pragmatic and theoretically flexible approach to addressing questions with implications for clinical practice and care.

### Participants and recruitment

Recruitment took place across participating hemodialysis units approximately 6–12 months after intervention rollout. Eligible participants included adult patients receiving intermittent hemodialysis for > 3 months, their informal caregivers or family members, and clinicians involved in hemodialysis patient care and symptom management (i.e., nurses, nephrologists, and allied health professionals [social workers, dietitians, spiritual care practitioners, kinesiologists]). We used purposive sampling with maximum variation to sample participants across clinic-demographic characteristics and type and extent of exposure to PROMs. A research coordinator approached eligible patients and clinicians in person during scheduled hemodialysis sessions and arranged an interview with those expressing interest. Eligible caregivers were identified by the corresponding patient and contacted only if the patient agreed and provided contact information. All participants provided oral or written informed consent.

### Data collection

A research coordinator (SL) experienced in qualitative interviewing undertook semi-structured interviews lasting 20 to 60 min with consenting participants. Patient interviews were completed in person during hemodialysis sessions, and health care provider interviews were completed in person, by telephone, or virtually, depending on availability. No repeat interviews were undertaken. Participants were asked about their experiences with the PROM intervention and their perspectives on integrating PROMs into routine hemodialysis care (Additional File 1). This included how they used the symptom assessment tools (e.g., PROMs, treatment guides) and how individual and environmental factors may have influenced PROM uptake. The interviewer summarized and reviewed responses with participants throughout the interview, but formal member checking was not undertaken. Interviews were audio-recorded, transcribed verbatim, and entered into NVivo 12 to facilitate data management, coding, and retrieval [31].

Two research team members, including a research coordinator (SL) and a patient with lived experience of kidney disease who contributed to the *EMPATHY* project team (not as a study participant) (BLC), conducted independent observations of PROM assessments and reviews during routine hemodialysis sessions in 6 distinct units. They documented field notes to capture contextual data about the hemodialysis setting and patient-provider interactions to supplement interview data. Observations were conducted upon expressed agreement by both the clinician and patient involved in the interaction.

### Analysis

We used reflexive thematic analysis appropriate to our study methodology to analyze interview and observational data [32]. This approach is suitable for qualitative research orientations where meaning is contextual and acknowledges the active role of the researcher in the process [33]. Analysis was inductive, or ‘data driven’, in that codes were developed to represent patterns of meaning as communicated by participants rather than fit into an existing coding framework [34]. Transcripts were distributed across research team members (MJE, SL, BHB), who coded them iteratively and in duplicate [33, 35]. The three team members generated a preliminary coding scheme through reviewing, coding, and discussing the initial 3 patient and 3 clinician transcripts together. We then applied preliminary codes to subsequent transcripts in duplicate and revised and updated the evolving coding scheme through team discussion. After coding 15 interviews, we had established our final codes and applied them to remaining transcripts. Coded extracts were compared across team members, and discrepancies were resolved through consensus to ensure analytic credibility. Research team members generated preliminary themes, which were refined through discussion and verified against the dataset to identify patterns and relationships. We analyzed field notes by applying the codes generated from our transcript analysis, which we also discussed and revised during team meetings. Field note analysis complemented and enhanced thematic findings emerging from interview data. Final themes were reviewed for consistency and coherence. Data collection and analysis took place simultaneously, and recruitment ceased once code saturation had been attained (i.e., when no additional concepts emerged and the coding scheme had stabilized) [36].

### Rigor and reflexivity

The research team includes researchers, clinicians, and a patient partner with a variety of academic backgrounds and lived experiences. The research coordinator leading interviews (SL) is a woman with a Master of Science degree in Speech-Language Pathology and several years of qualitative research experience with the research team. The study’s lead investigator (MJE) is a nephrologist and clinician-scientist with qualitative research expertise and an interest in the topic area. Other team members drew on their experiences as clinicians (i.e., nephrologist, nurse, physiotherapist, pharmacist), researchers in the content and methodological areas, and patients with lived experiences of kidney failure when interpreting our study findings. Team members documented reflexive notes throughout analysis. Those involved in data collection had no prior knowledge of study participants and were not involved in the clinical care of people receiving hemodialysis or PROM administration. Patient engagement in this study was guided by strategies and guidelines of the Can-SOLVE CKD patient-oriented research network [37]. We took steps to ensure rigor and trustworthiness of our study [38] and have reported our study in accordance with the Consolidated Criteria for Reporting Qualitative Research (COREQ) reporting standards [39]. Our study was approved by the University of Calgary’s Conjoint Health Research Ethics Board (REB18-1786).

## Results

We completed 54 interviews with 33 clinicians (22 nurses, 6 nephrologists, 5 allied health professionals), 20 patients, and one family member of a patient who did not participate (Tables 1 and 2). Only 5 eligible individuals that we approached declined participation due to lack of interest. Approximately two-thirds of patients were male, and one-third had been on dialysis for > 5 years. Of the 33 clinicians, most were female and had held their current role for ≤ 10 years. We completed a total of 19 observations of PROM assessments (13 conducted by a research coordinator and 6 by a patient partner).

**Table 1:** Patient and caregiver characteristics (n = 21)

Socio-demographic characteristic	N (%)
*Gender*	
Man	13 (61.9)
Woman	8 (38.1)
*Age (years)*	
Under 40	1 (4.8)
40–64	13 (61.9)
65 or older	7 (33.3)
*Education*	
Less than Grade 12	1 (4.8)
High school diploma	7 (33.3)
College, trade, university	13 (61.9)
*Employment*	
Retired	8 (38.1)
Disability	6 (28.6)
Not employed	5 (23.8)
Employed full or part time	2 (9.5)
*Primary hemodialysis location (population)*	
Large urban (100,000 and over)	10 (47.6)
Medium urban (30,000–99,999)	4 (19.1)
Small urban (1000–29,999)	7 (33.3)

Clinical characteristic*	N (%)
*Cause of kidney failure*	
Diabetes	6 (28.6)
High blood pressure	1 (4.8)
Glomerulonephritis	1 (4.8)
Other (e.g., sepsis, obstruction)	12 (57.0)
Unknown or unsure	1 (4.8)
*Length of time with kidney disease (years)*	
Less than 5	7 (33.3)
5–9	6 (28.6)
10–20	5 (23.8)
More than 20	3 (14.3)
*Length of time on hemodialysis (years)*	
Less than 1	6 (28.6)
1–2	5 (23.8)
3–5	3 (14.3)
More than 5	7 (33.3)
*Experience with other kidney failure treatments*	
Yes	7 (33.3)
Peritoneal dialysis	4 (19.1)
Home hemodialysis	2 (9.5)
Transplant	1 (4.8)
No	14 (66.7)
*PROM allocation*	
EQ-5D-5L	9 (42.9)
IPOS-Renal	8 (38.1)
Both	4 (19.0)

IPOS, Integrated palliative care outcome scale; PROM, patient-reported outcome measure

*The one participating caregiver reported clinical characteristics of her spouse who did not participate in the study

**Table 2:** Healthcare provider characteristics (n = 33)

Socio-demographic characteristic	N (%)
*Role*	
Nurse	22 (66.7)
Nephrologist	6 (18.2)
Allied health	5 (15.2)
*Gender*	
Woman	27 (81.8)
Man	6 (18.2)
*Age (years)*	
Under 40	13 (39.4)
40–64	19 (57.6)
65 or older	1 (3.0)
*Education*	
Undergraduate degree	16 (48.5)
College diploma	8 (24.2)
Professional degree	6 (18.2)
Graduate school	3 (9.1)
*Employment*	
Full time	18 (54.5)
Part time/casual	15 (45.5)
*Primary work location (population)*	
Large urban (100,000 and over)	26 (78.8)
Medium urban (30,000–99,999)	3 (9.1)
Small urban (1000–29,999)	4 (12.1)
*Time in current role (years)*	
5 or less	10 (30.3)
6–10	11 (33.3)
11–15	4 (12.1)
16 or more	8 (24.3)

**Fig. 1:**
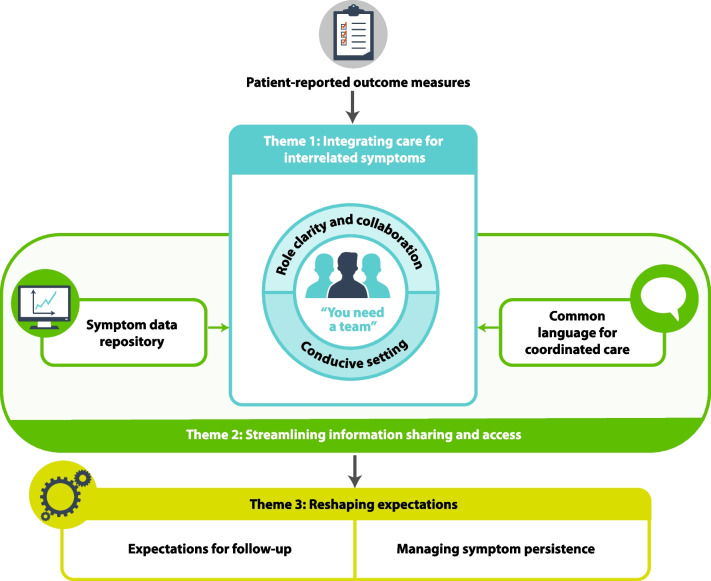
Relationship between thematic findings. PROM use supported an interdisciplinary approach to symptom management within the hemodialysis unit by highlighting the interrelatedness of symptoms and helping define team members’ roles and responsibilities to address patients’ evolving needs (Theme 1, *blue*). PROM use established a symptom data repository and streamlined communication channels that influenced interprofessional and patient-provider interactions (Theme 2, *green*). When symptoms persisted, PROMs prompted broader conversations between patients and their care team about illness perception and realistic goal setting (Theme 3, *yellow*)

Despite initial unfamiliarity with PROMs, clinicians discussed how they used them to coordinate care across provider types and address patients’ complex and multi-faceted needs. Patients tended to focus more on the perceived impact of concerted care resulting from PROM use on their symptom trajectories and dialysis-related experiences. Participants across roles identified patients as integral members of this interdisciplinary team and discussed the utility of PROMs in interdisciplinary symptom management in relation to three overarching themes: (1) Integrating care for interrelated symptoms; (2) Streamlining information sharing and access; and (3) Reshaping expectations. Figure 1 demonstrates the relationship between themes and processes by which PROMs may strengthen interdisciplinary hemodialysis care. Additional quotes in support of themes and subthemes are presented in Table 3.

**Table 3:** Additional selected participant quotes supporting identified themes and subthemes

Theme		Illustrative quote
*Integrating care for interrelated symptoms* Patients’ high symptom burden, complexity, and interrelatedness are well suited to an interdisciplinary approach. The hemodialysis unit can serve as a hub to coordinate interdisciplinary care given its convenience, familiarity, and access to comprehensive kidney care resources and personnel. Interdisciplinary team members assume defined yet fluid roles in addressing concerns identified by PROMs and their underlying physical and psychosocial contributors		
“You need a team”	*"I did see a patient who all of them [symptoms] were moderate to severe, and I said, ok this patient must not have understood the survey, must not have done it; however, when I spoke to them it was true. And that*’*s the sad part. And the worst part is, I cannot pick three [symptoms]. The patient can't even pick three. I had to go through everything, but it turned out they are all correlated.”* (Nurse 10) *“[Kinesiologist] will deal with the pain, fatigue, poor mobility, and the restless legs, because she tries to use exercise to help the patient… Let’s say if the patient [has] the pain relieved, then maybe the patient has better sleeping. And maybe if the pain is relieved, the mobility is better.”* (Nurse 20) “*You are lucky if you have a pharmacist, only certain units have a pharmacist… So that*’*s kind of sad. Our team is pretty small. It*’*s really just the charge nurse, bedside nurse, and patient.” (Nephrologist 6)* *“Even with EMPATHY, you can't go and grab every symptom. Those are the main 13 [symptom] questions. There*’*s a lot more that goes on in life of the person with kidney disease. Like, I have all the top three symptoms, sometimes I don*’*t have the symptoms, sometimes they come back.”* (Patient 3)	
Conducive setting	*“They [patients] do trust us with their whole life, so they will tell us things that they will tell nobody else. I think because we are given that confidence from them, then we take that on for them.”* (Nurse 12) *“As for the [EMPATHY] surveys, I think the best time to do them is when you are on dialysis because a lot of times you are looking for something to do anyways.”* (Allied health professional 2) *"If I have an issue on the weekend and I’m coming here on Tuesday, I don’t even call my GP [general practitioner], I just come here. I know there’s going to be a doctor coming around, and if he says to do follow-up with your GP, then I’ll do it. A lot of times I can get good answers right here.”* (Patient 1) *“It’s all electronic, and that’s how we are communicating. So I’ve been totally involved, I’ve got binders and all my blood work and all that stuff, I’ve got a paper copy… I take [my symptom reports] to my family doctor for example… I’ve shown him that, and it’s above his head… he’s overwhelmed by kidney disease, he doesn’t get it.” (Patient 3)* *“I tell them that I appreciate their [dialysis staff] opinion because it’s good to hear someone who has experienced it and works here at the same time. It’s a kidney clinic. Rather than listening to an outsider that has not experienced it, they have experienced it, but at a different level.”* (Patient 12)	
Role clarity and collaboration	*“I found it quite interesting the similar trends that came out in all the patients, and especially a high focus on the anxiety and depression that we don*’*t really hit on that much. It opened a lot of dialogue among the staff and on our different committees about what we can do better about that.”* (Nurse 1) *“We are not just the nurses here. We are the mini social workers, we are the mini dietitians… we are mini technicians when the machines break. We have to do a lot of stuff I think that maybe in bigger centres, you have individuals for these jobs.”* (Nurse 8) *“I truly believe in my everyday nursing, I try to look at the patient holistically. When you see them frequently then you can assess whether things have changed, like emotionally or physically and then with bloodwork. So something that we are doing all the time.”* (Nurse 18) *“The nurses have gotten stronger in their ability to present the patient’s case to the rounding physician. They are more comfortable with it than they used to be… The individual nurse providing care is speaking to and using the rounding tool for that purpose and I think the EMPATHY [symptom] reports and this whole approach is strengthening that.”* (Allied health professional 3)	
*Streamlining information sharing and access* Serial capture of PROMs creates a central repository of patient-focused information to which interdisciplinary team members can refer for surveillance of patients’ concerns and responses to therapy over time. Accessibility of PROM data across sites and providers helps to streamline processes for escalating and documenting care approaches. Visual and tangible resources (e.g., PROM reports, treatment guides) can be integrated into care discussions with patients and between clinician team members		
Symptom data repository	*“Sometimes we miss some parts of the information, but those EMPATHY reports, they have everything, so it’s easy for us too.”* (Nurse 3) *“We do put on there [rounding tool] what we have given them and also chart it in [electronic health record], and then hopefully whoever has given the handout will indicate that and then you say, ‘Oh, I see you’ve been given a handout. Did anything on that help?’”* (Nurse 16) *“I know that now at least we have access to [symptom reports and handouts], because every single computer now has that link, so I think that helps.”* (Allied health professional 4) *“There*’*s going to be consistent notes of what*’*s worked and tried, has been trialed and failed… I think EMPATHY is so important and those issues are probably the most important issues to our patients. I’m sure they could care less what their phosphate is, but I don*’*t think you can make significant differences unless you have some consistency and some communication between practitioners.”* (Nephrologist 4) “*No, I haven*’*t [seen a symptom report], at least I don*’*t think I have.”* (Patient 20*)*	
Common language for coordinated care	*“I think maybe [I am] charting more, and I*’*m getting a lot more referrals, which is great, from the nursing staff. That*’*s changed a lot.”* (Allied health professional 1) *“They were marking down what handouts the patients got, and I think that was supposed to trigger other team members so that they could follow-up with them if they felt they needed to.”* (Allied health professional 2) *“They use the resources. They use pharmacy. They use social work. Not all patients have that [internal drive]. I think we need to give them the language, because if you give that to them and teach them how to speak that… common language, right? ‘What are your stop signs?’, ‘What would you like to do today?’”* (Nurse 12) “*We are referring right away. If they are weak, right away we refer to the kinesiologist, she*’*s following up. Anything to do with anxiety or depression, I refer them to the social worker. For any of those nauseous, [I] refer to dietician and they email me back right away and say, yes, I*’*ll see them tomorrow… It*’*s been good.” (*Nurse 19*)* *“Even though they can give me a tip sheet, most of that stuff I*’*ve already tried it. Been there, done it. That*’*s when the communication comes in with the nurse that*’*s there. I don*’*t know if that*’*s happening in other units, I’m not there. I can see that happening [here].”* (Patient 3)	
*Reshaping expectations* Patients’ disclosure of symptoms and concerns using PROMs leads to expectations of treatment, follow up, and observed improvement. The chronic and complex nature of kidney failure means that symptoms often persist despite appropriately escalated therapy. In such circumstances, the interdisciplinary team plays an important role in validating concerns, reframing illness expectations, and assisting patients and their loved ones to develop coping strategies to live well		
Expectations for follow-up	*“It takes all this time and effort and you are doing the survey one-on-one with the patient and then you have to chart it all and then go through all the teaching and the handout sheets and everything, and then nothing gets done.”* (Nurse 14) *“If you have that [symptom] going on for, never mind two or three months, maybe even just two or three weeks, I*’*m sure the patient, they just throw up their hands and say, ‘Thanks for asking me about my symptoms, but what*’*s the point because no one is doing anything about it?’”* (Nephrologist 2) *“It’s a team process, and the patient is at the centre… Maybe [patients] have some ‘concern fatigue’. Maybe every week they tell people what their concerns are and nothing ever happens, so pretty soon they stop bringing it forward. Hopefully EMPATHY has made that a little bit better.”* (Nephrologist 6) *“We do get asked every run if we [have] nausea, vomiting, diarrhea, any pain, bleeding, or falls, which are obviously good questions. Necessary to know. But I think that I wouldn’t have the conversation unless it was something that was really bothering me, and then I don*’*t know that anything really becomes of it… I feel like the nurse maybe thinks that I’m just venting.”* (Patient 6) *“The surveys are great. I think the survey helps everybody,* if *they listen to it.”* (Patient 13)	
Managing symptom persistence	*“People just want to be heard as well, and it’s communicating with them. I think a lot of apprehension is not knowing what to expect… When you talk through things, that helps alleviate a lot of anxiety, that what they are experiencing is where they are going to be at. And, bit by bit, it will improve, because the thing that I got from him [patient] was he wasn’t really given the true expectation. We see that all the time.”* (Nurse 6) *“We do a bit of expectation management, maybe not CBT [cognitive behavioural therapy] *per se*, but readjusting our mental state to attack the problem from a different angle or to recognize the limitations of dialysis.”* (Nephrologist 4) *“Commiseration is not helping you. Thinking, ‘Why me?’, is not going to help you. You have to be [an] optimist and believe that something is going to be better tomorrow or later on… because if the [clinicians] are treating them the way they are treating me, there is no way to for them to feel so bad.”* (Patient 4) *“For example, some days I have really bad bone pain and I am already doing all of the things that I can do to alleviate that. There*’*s been suggestions that are always welcome, but sometimes this is just a problem I have that nobody can really do anything more for.”* (Patient 6)	

### Integrating care for interrelated symptoms

#### “You need a team”

In addition to identifying the presence of symptoms, participants described how PROMs underscored symptom complexity and interrelatedness. The co-occurrence of physical symptoms (e.g., nausea, decreased appetite, constipation) with each other and mental health concerns (e.g., depression, anxiety) were linked to similar root causes.

*"There is a lot of interaction or overlap in those concerns. If the person’s not sleeping and has restless legs or pruritus, there is a good chance that they are going to be depressed… It is also a symptom of living with a chronic illness.”* (Allied health professional 3)

Patients and clinicians explained how it was neither realistic nor appropriate to expect any one health professional to address patients’ complement of symptoms. They also identified how competing health and life demands for people receiving hemodialysis posed additional management challenges that would benefit from a team-based approach. They discussed integrating PROMs with symptom management tools as a way of tackling concerns from different angles, provided hemodialysis units were equipped with the necessary resources and personnel.

*“You need a team… I think that’s even more important when it comes to the types of issues that EMPATHY is dealing with.”* (Nephrologist 4)

#### Conducive setting

Patients and providers noted the conduciveness of the hemodialysis setting to integrated symptom management using PROMs. Not only was the environment familiar to patients, but it enabled longitudinal surveillance and tailoring of care plans during scheduled hemodialysis sessions. Patients and the participating caregiver described developing strong rapport over time with their hemodialysis care team, and providers relayed how previously established relationships with patients and colleagues enabled integrated symptom management using PROMs. This familiarity and comfort among patients and caregivers and their care team was reinforced through rapport, clear communication, and collegiality, as noted during most observations of PROM-related interactions in the hemodialysis unit.

*“They [dialysis nurses] are always very receptive to answering my questions or giving any information if there is an issue*.” (Caregiver 1)

Although the PROM initiative centered on hemodialysis care, few patients had referred to their symptom reports outside of the hemodialysis unit, such as during encounters with family physicians. Clinicians anticipated that PROMs could be used to engage members of the extended care team, including community health professionals, in symptom management but lacked guidance for doing so.

*“I’ve been totally involved, I’ve got all my blood work and all that stuff, I’ve got a paper copy… I take [my symptom report] to my family doctor, for example… I’ve shown him that, and it’s above his head… He’s overwhelmed by kidney disease, he doesn’t get it.”* (Patient 3)

#### Role clarity and collaboration

Clinicians described using PROMs to delegate team members to address issues within their scope of expertise. They explained how bedside nurses first reviewed patients’ PROM reports to identify symptomatic concerns, which often triggered focused assessments by allied health professionals and use of treatment guides (i.e., treatment algorithms for patients and clinicians) to direct initial management of the main concerns identified by PROMs. The interrelated nature of symptoms meant that several providers were often needed to address symptoms and their contextual contributors, such as external supports or financial constraints.

*“I really liked the content [of treatment guides]. I liked how it broke up into… what the nurse can do, what the kinesiologist, pharmacy people can do, what the physician needs to do.”* (Nurse 1)

Symptom management protocols relied on hemodialysis units having access to suitable expertise to address the wide variety of health concerns identified by PROMs. Rural clinicians, in particular, described reduced access to some specialized resources and relying on role versatility to offset these challenges. Some nurses discussed the centrality of holistic care to the nursing philosophy and how PROMs could either reinforce or undermine this purpose, depending on how they were used during patient encounters—whereas some appreciated additional opportunities to engage in symptom-focused discussions with patients and enhanced role fluidity, others suggested that use of PROMs as screening checklists without meaningful interaction could unnecessarily systematize a process that already takes place more organically. Nurses and nephrologists acknowledged the importance of symptom management, but several suggested they prioritized their obligation to ensure safe and adequate dialysis and oversee its technical aspects.

*“Now [treatment guides] give the nurses an extra tool in their armour… and if [patients] are still not happy then they can always come back and say, ‘Can I talk to a doctor?’”* (Patient 2)

### Streamlining information sharing and access

#### Symptom data repository

Clinicians explained how formalized capture of symptom trends using PROMs enabled information sharing between team members and with patients. They indicated that electronic documentation of symptoms and attempted therapies helped establish a central repository of patient-focused data that could be accessed longitudinally and across sites and providers. Despite the potential for increased charting burden imposed by PROMs, harmonized documentation was emphasized as a way of promoting efficient information exchange.

*“I would like to see more of that general communication amongst the healthcare providers, if they’ve already talked to the patient or already given the [symptom] handout.”* (Allied health professional 2)

Symptom reports generated from PROMs were maintained in patients’ paper and electronic records to provide an accessible and visual means of tracking symptom trends. Some patients indicated they retained copies of these reports for their own records. Nurses described annotating reports to facilitate review with patients, which was corroborated during field observations, and suggested their availability in multiple formats and locations helped bring patients’ concerns to the attention of different providers, such as the rounding nephrologist.

*“If the survey is physically sitting on the chart and I can see what the patient’s marked off, then maybe I can get a clue as to what the patient is interested in.”* (Nephrologist 2)

Most patients and the participating caregiver appreciated reviewing their symptom trends but noted that these were shared inconsistently (i.e., at irregular times or sometimes not at all). Some patients who described lower engagement in the initiative did not recall having reviewed their symptom reports with their care team.

#### Common language for coordinated care

Participants across roles suggested that PROM use facilitated interdisciplinary team engagement in a purposeful, targeted, and proactive fashion. Because symptom reports and care plans were readily accessible, allied health professionals described reviewing and discussing available patient data to anticipate a need for their involvement prior to formal consultation.

*"If symptoms are persisting, usually there’s a next step of a referral to allied health, but because each of us have access to the patient’s [symptom] report on our own, especially when there’s a relationship already there, I will often follow up.”* (Allied health professional 5)

In addition to equipping patients and staff with common terminology (e.g., “stop sign”, “symptom report”), clinicians indicated that familiarization of patients’ symptoms using PROMs helped guide discussions, identify therapeutic priorities, and direct patients to resources at the point of care, such as printable handouts on bedside computers. Clinicians highlighted the utility of PROM-related documentation in cataloging the involvement of various services in patient care, which promoted transparency and reduced redundancy. Some patients noted increased accessibility to allied health services since PROM use began in their centres.

*“Lately, it’s easier to reach the services that we have here, because it felt difficult before. The pharmacist is great and you can talk to her whenever. Social worker is great, you can make an appointment with her… I think it’s maybe their awareness of the EMPATHY project.”* (Patient 6)

However, several allied health professionals noted unintended consequences of increased referrals and anticipated difficulties in managing caseloads resulting from higher identified symptom burden using PROMs.

### Reshaping expectations

#### Expectations for follow-up

Patients and clinicians indicated how the use of PROMs to capture and initiate conversations about patients’ concerns was an important first step to addressing the issues affecting their health-related quality of life. This was notable for issues that some considered sensitive (e.g., mental health concerns) or that patients may not have raised without prompting. Disclosure by patients was accompanied by confidence in follow-up by the interdisciplinary team. Patients and clinicians expressed concern that this confidence could be undermined if identified issues were not reviewed with patients in a timely manner or given appropriate attention.

"*I wouldn’t have the conversation unless it was something that was really bothering me, and then I don’t know that anything really becomes of it." (Patient 6)*

Clinicians described how awareness of patients’ expectations for follow-up accompanying PROM use was a motivating factor for person-centered, collaborative care, as it reinforced the purpose of the initiative.

*"Out of respect to the patient, we really owe it to them, if they take the time to do the survey and divulge this information, that we are diligent in following up with it.”* (Allied health professional 2)

#### Managing symptom persistence

Patients and clinicians explained how serial completion of PROMs often highlighted symptom persistence, despite care escalation according to treatment protocols. While non-resolving symptoms varied across patients, patient and clinician participants related them to a lack of easily identifiable solutions or triggers (e.g., fatigue). They appreciated tracking symptoms month to month to bring awareness to areas of improvement, persistence, or worsening.

*“[It] always is good to know what you can expect from this kind of illness, because when you don’t know, something can scare you, and that is not good.”* (Patient 4)

Patients related their frustration with symptom persistence to perceived treatment ineffectiveness and lack of available therapeutic strategies. Once suggested treatments had been exhausted, clinicians expressed uncertainty about next appropriate steps or services to engage. Several observed interactions in hemodialysis units centered around issues that persisted across serial PROM reports. Clinicians proposed using such scenarios to validate patients’ concerns and engage the interdisciplinary team in managing expectations around symptom persistence.

*I think it’s probably good for people’s mental health just to be heard… To validate their concerns. We are trained to be fixers, and fixing isn’t always the answer.”* (Nephrologist 4)

Several clinicians explained how concerted efforts across physician, nursing, and allied health colleagues were necessary not just to treat symptoms identified by PROMs, but to provide structured support when symptoms endured. They suggested open communication, coping, and goal setting as strategies to align care expectations of patients and providers and help reframe illness perception.

*"Even if the symptoms don’t go away, if their perception of overall health [is] improving, that’s really important too.”* (Allied health professional 5)

## Discussion

In this study, we characterized patients’ and clinicians’ perspectives on the role of PROMs in supporting interdisciplinary symptom management for people receiving hemodialysis. Across three overarching themes, participants highlighted how the pervasiveness and interrelatedness of symptoms necessitated an interdisciplinary, team-based approach that included patients. Established relationships and resources within the hemodialysis unit made this a conducive environment for using PROMs to identify symptomatic concerns, communicate care plans, and define roles and responsibilities to meet patients’ evolving needs. Capture of symptom data through PROMs was met with an expectation for timely follow-up and management. Under circumstances of symptom persistence, PROMs prompted broader conversations between patients and their care team about illness perception and realistic goal setting.

The symptoms that patients undergoing hemodialysis consider most debilitating are often multi-factorial, with physical, psychological, and socioeconomic contributors [1]. These symptoms can be difficult to target in isolation and thus are optimally addressed through comprehensive, team-based care, where providers integrate their expertise with patients’ priorities to tackle issues from different angles [40]. In other settings where symptom control and health-related quality of life are therapeutic mainstays, such as palliative care, an interdisciplinary approach can improve physical and mental wellbeing [41]. In nephrology, multidisciplinary clinics for people with non-dialysis-dependent chronic kidney disease have been associated with delayed disease progression and lower mortality [21, 22, 42]. Whereas multidisciplinary care refers to clinicians with distinct expertise working in parallel to one another, our findings support enhanced interdisciplinary care through collaborative goal setting and care integration across disciplines using PROMs [20, 43]. Our findings also include instances suggestive of an extension toward transdisciplinary care, whereby role fluidity and flexibility allowed clinicians to provide care outside of their traditional disciplinary scope (e.g., rural nurses providing dietary or exercise counselling) [44, 45].

In the hemodialysis setting, ill-defined roles among healthcare providers have been cited as a barrier to effective symptom management [11]. Time and resource constraints can also limit what is achievable during patient-clinician encounters. For example, the short average duration of interaction between a patient and the rounding nephrologist is likely insufficient to address the complex issues raised by patients [46, 47]. Moreover, hemodialysis clinicians may believe it is not within their purview to address issues such as depression or chronic pain [11, 48–50]. This was corroborated by some of our study’s clinician participants, who described prioritizing dialysis safety and adequacy over subjective concerns, and by patient participants, who described reluctance in raising certain issues with their hemodialysis providers. Allied health professionals appreciated the streamlined referral processes resulting from PROMs, but their concerns about increasing caseload and resource constraints raise considerations about how to extend interdisciplinary symptom management beyond the hemodialysis unit. Integrated care models that engage primary care and other community health resources in kidney care delivery could mitigate some of these concerns [51], although few patient participants said they had discussed their PROM reports during encounters with non-dialysis clinicians. This application requires further study.

Processes to support patient-provider and interdisciplinary communication, such as clinical rounding tools, can enable concerted care for people receiving hemodialysis [52]. In a report by Dorough et al., an interdisciplinary plan-of-care program with components of team education, patient collaboration, and action planning enhanced patient care experience and encouraged a more individualized, person-centered approach [53]. In our study, participants discussed how PROMs complemented existing patient assessment structures by providing consistent, reliable, and objective symptom documentation, which helped team members familiarize themselves with patients’ symptom profiles and proactively engage in their care. This approach was received positively by many, but not all, patient and clinician participants, which is consistent with the mixed influence of PROMs on hemodialysis team communication reported in another study [54].

Our findings underscore a potential for PROMs in hemodialysis units to promote person-centered care, which refers to coordinated, responsive care that prioritizes individuals' clinical, social, emotional, and practical needs [55]. Despite documented benefits of person-centered care, including improved patient care experience and health outcomes, much of routine hemodialysis care focuses on its technical, physiological, and medical aspects (e.g., dialysis adequacy, blood pressure control, anemia targets) reflected in traditional disease-centered care models organized for the convenience of providers [56, 57]. In our study, PROMs provided dedicated opportunities for patients to prioritize their concerns and share patient-sourced symptom data with their care team. With this came the surfacing of symptoms that are often overlooked in this setting, such as mental health concerns, raising important questions about how routine PROM use may be optimized to meet patients’ physical and psychological support needs [50, 58].

Participants underscored the need for appropriate action in response to symptom information shared with the dialysis care team through PROMs, as identified in another study [49]. In this way, PROMs can serve as both a prompt to follow up with patients and a means of tracking their symptom trajectories and treatment responses over time. For some patients, however, symptoms, such as fatigue, persist despite escalated therapy and attempts to address their root causes [59]. Thus, our findings suggest another important application of PROMs in the interdisciplinary management of symptom persistence, where they can inform discussions between patients and clinicians, help establish realistic expectations, and redirect the focus of care to illness perception and coping. Similarly to Dorough et al.’s structured plan-of-care program, our findings support the need to align patient and provider priorities and individualize care, but using PROMs to mirror patients’ evolving symptomatic needs [53].

Our study has several strengths, including its sampling breadth and patient partner engagement; however, we acknowledge some limitations. All participants were sampled from hemodialysis units in Southern Alberta, where aspects of care (e.g., hemodialysis rounding procedures, resource availability) and methods used to implement PROMs may differ from other programs. Our study also took place alongside staggered rollout of the larger *EMPATHY* initiative, which meant that participants across eligible hemodialysis units may have had varying familiarity and comfort with using PROMs. However, we sampled participants across settings in our program (e.g., urban/rural) and approached eligible individuals halfway through the one-year initiative to permit sufficient experience with the PROM intervention. Although we report only on the perspectives from interested individuals who consented to study participation, our purposive sample across a breadth of clinical and demographic characteristics was intended to reflect the more broadly eligible population. As nearly all patients declined extension of our study invitation to their caregivers, we interviewed only one who provided a complementary perspective that largely reinforced patients’ responses. Lastly, we acknowledge that interviews conducted during hemodialysis sessions were of shorter duration and often not private, and thus may have influenced disclosure. Participants were offered alternative interview formats, and those wishing to proceed may have chosen this format for their own comfort or convenience. Other studies have used similar interview approaches during hemodialysis without compromising data quality [49, 60, 61]. Future research should explore the implications of PROM use for caregivers, application of the study’s PROM resources outside of the hemodialysis setting (e.g., with patients’ primary care physicians), and preferences for and influence of different PROM types on interdisciplinary hemodialysis care.

## Conclusion

In the context of a program-wide initiative to integrate PROMs into routine hemodialysis care, we found that PROMs underscored symptom interrelatedness and complexity and helped to streamline involvement of the interdisciplinary care team to address symptomatic concerns from different vantages. Whereas some clinicians identified opportunities to expand their traditional roles to meet patients’ evolving needs, others pointed to resource and capacity constraints that could affect sustainable use of PROMs to promote interdisciplinary symptom management in this setting. Symptom pervasiveness in this population highlights an important use of symptom reports in guiding illness conversations and helping reframe care expectations.

## Supplementary Information

**Additional file 1**. Interview guide for patients, caregivers, and clinicians.

## Data Availability

We are unable to make our dataset available due to potential identifiability of participating individuals from our qualitative data. De-identified data may be made available from the corresponding author upon reasonable request.

## Abbreviations

Can-SOLVE CKD: Canadians seeking solutions and innovations to overcome chronic kidney disease
COREQ: Consolidated criteria for reporting qualitative research
EMPATHY: Evaluation of routinely measured PATient reported outcomes in HemodialYsis care
IPOS: Integrated palliative care outcome scale
PROM: Patient-reported outcome measure
