# Supplementary material for: “You need a team”: perspectives on interdisciplinary symptom management using patient-reported outcome measures in hemodialysis care—a qualitative study
Source: J Patient Rep Outcomes. 2023 Jan 20;7:3. doi: 10.1186/s41687-022-00538-8 (PMC9859959; doi:10.1186/s41687-022-00538-8)
Supplement: Supplementary file 1 — Additional file 1. Interview guide for patients, caregivers, and clinicians. [file 41687_2022_538_MOESM1_ESM.docx]

**Additional file 1.** Interview guide for patients, caregivers, and clinicians.

| **Patients/Caregivers** | **Clinicians** |
| --- | --- |
| **PROM completion** | |
| What has it been like completing the PROM surveys every 2 months?   - Do questions cover all symptoms that are important to you? Explain. - How did you discuss your concerns previously with your nurse or doctor? How does this compare? | Tell me about your experience with the PROM surveys every 2 months.   - What do you think about the format and content? - Compare how you previously identified patient concerns at dialysis with how they are identified now. |
| What has it been like receiving the information sheets?   - What do you think about the information on the sheets? - How were your concerns previously addressed with your nurse or doctor? How does this compare? | Tell me about your experience with the treatment aids (e.g., guidelines and information sheets).   - What do you think about the format and content? - How did you previously address concerns with patients? How does this compare? |
| **Setting and context** | |
| How do others support you in completing the surveys and addressing your concerns? (e.g., family members, friends, healthcare team)  Are there any other ways that your dialysis unit assists you? | How were the PROM surveys introduced and explained to you? (i.e., how surveys would be used as part of your regular job duties)  How do your manager and colleagues support you in completing the surveys and using treatment aids with patients? |
| What preparation/training did you receive to complete the PROM surveys? How useful was it? | What preparation/training did you receive to complete the PROM surveys with patients? How useful was it? |
| Tell me about any opportunities to provide feedback about the surveys and information sheets. | Tell me about any opportunities to provide feedback about the surveys and information sheets. |
| **Using PROM results** | |
| How do surveys and information sheets get incorporated into your care during a dialysis session?   - How are the surveys scheduled on your unit? - How are the information sheets reviewed with you?   How were you made to feel involved in this process? | How do you integrate the surveys and treatment aids as part of [interdisciplinary] patient care?   - How are the surveys scheduled on your unit? - How do you review the information sheets with patients?   How do you involve patients/other clinicians in this process? |
| How easy is it to complete the PROM surveys and/or review the information sheets during dialysis?  What difficulties do you have, if any? | How easy is it reviewing the report cards and information sheets during dialysis?  How much assistance do patients require to complete the surveys, if any? |
| What have you learned from doing these PROM surveys at dialysis? (e.g., about yourself, how to manage your health, how the care team helps you manage your symptoms)  How do you use the information provided to you about your health concerns? How do they address your needs? | What have you learned from doing these PROM surveys with patients (and from their responses)?  How useful are surveys and treatment aids to your practice? Explain.   - Do you think the surveys and treatment aids are helpful to patients? Why or why not? |
| **Overall** | |
| What suggestions do you have about how to best use these surveys in your dialysis unit? | What suggestions do you have about how to best incorporate these surveys and treatment aids into patient care in your dialysis unit? |
| Is there anything else you’d like to tell me? | Is there anything else you’d like to tell me? |
